# Discovery of a New CaMKII-Targeted Synthetic Lethal Therapy against Glioblastoma Stem-like Cells

**DOI:** 10.3390/cancers14051315

**Published:** 2022-03-04

**Authors:** Jang Mi Han, Yu Jin Kim, Hye Jin Jung

**Affiliations:** 1 Department of Pharmaceutical Engineering & Biotechnology, Sun Moon University, Asan 31460, Korea; gkswkdal200@naver.com (J.M.H.); petaldew17@naver.com (Y.J.K.); 2 Genome-Based BioIT Convergence Institute, Sun Moon University, Asan 31460, Korea

**Keywords:** synthetic lethal therapy, glioblastoma stem-like cells, calcium/calmodulin-dependent protein kinases II, neurokinin 1 receptor, hydrazinobenzoylcurcumin, berbamine, SR 140333, aprepitant

## Abstract

**Simple Summary:**

The poor prognoses of patients with glioblastoma multiforme (GBM) are attributed to glioblastoma stem-like cells (GSCs), which induce drug resistance and rapid tumor recurrence. Accordingly, the discovery of promising therapies targeting GSCs is important for the treatment of GBM. The purpose of this study is to explore a novel synthetic lethal therapy targeting calcium/calmodulin-dependent protein kinase II (CaMKII), an emerging target for combating GSCs. Through high-throughput drug combination screening using CaMKII inhibitors and a library of bioactive compounds in GSCs, we find that the combined treatment with CaMKII and neurokinin 1 receptor (NK1R) inhibitors exhibit chemical synthetic lethal effects both in vitro and in vivo. Further analyses at the molecular level demonstrate that NK1R is a potential synthetic lethal partner of CaMKIIγ in its role in eradicating GSCs. Therefore, we propose a novel CaMKII-targeted combination therapy for the effective treatment of GBM.

**Abstract:**

Glioblastoma stem-like cells (GSCs) drive tumor initiation, cancer invasion, immune evasion, and therapeutic resistance and are thus a key therapeutic target for improving treatment for glioblastoma multiforme (GBM). We previously identified calcium/calmodulin-dependent protein kinase II (CaMKII) as an emerging molecular target for eliminating GSCs. In this study, we aim to explore a new CaMKII-targeted synthetic lethal therapy for GSCs. Through high-throughput drug combination screening using CaMKII inhibitors and a bioactive compound library in GSCs, neurokinin 1 receptor (NK1R) inhibitors such as SR 140333 and aprepitant are found to be potential anticancer agents that exhibit chemical synthetic lethal interactions with CaMKII inhibitors, including hydrazinobenzoylcurcumin (HBC), berbamine, and KN93. Combined treatment with NK1R and CaMKII inhibitors markedly suppresses the viability and neurosphere formation of U87MG- and U373MG-derived GSCs. In addition, the combination of HBC and NK1R inhibitors significantly inhibits U87MG GSC tumor growth in a chick embryo chorioallantoic membrane (CAM) model. Furthermore, the synthetic lethal interaction is validated using RNA interference of CaMKIIγ and NK1R. Notably, the synthetic lethal effects in GSCs are associated with the activation of caspase-mediated apoptosis by inducing p53 expression and reactive oxygen species generation, as well as the suppression of stemness marker expression by reducing nuclear factor-kappa B (NF-κB) activity. This follows the downregulation of phosphoinositide 3-kinase (PI3K)/protein kinase B (AKT) signaling and a decrease in intracellular calcium concentration. Moreover, NK1R affects CaMKIIγ activation. These findings demonstrate that NK1R is a potential synthetic lethal partner of CaMKII that is involved in eradicating GSCs, and they suggest a new CaMKII-targeted combination therapy for treating GBM.

## 1. Introduction

Glioblastoma multiforme (GBM), which the World Health Organization (WHO) classifies as a grade IV glioma, is a type of human primary brain tumor with the highest degree of malignancy and the worst prognosis [1]. Surgical resection followed by adjuvant chemoradiation therapy with temozolomide (TMZ), a DNA alkylating agent, is the standard treatment for GBM [2]. Despite multimodal therapeutic interventions for GBM, the average survival time of patients with GBM is estimated to be less than 16 months [2,3]. Food and Drug Administration (FDA)-approved TMZ has been used as a first-line chemotherapeutic agent for the treatment of GBM since 2005; however, chemotherapy with TMZ increases the average survival by only 2.5 months and eventually causes resistance to the drug in most cases [2,3]. The high mortality rate of GBM is due to its aggressive features, including accelerated growth, deregulated apoptosis, and invasion of surrounding tissues [4]. The main causes that confer drug resistance in GBM include drug efflux, DNA damage repair, tumor-induced hypoxic areas, microRNAs (miRNAs), and cancer stem cells [4,5,6]. Glioblastoma stem-like cells (GSCs), one of these causes, are generated in GBM and play a crucial role in tumor initiation, cancer invasion, immune evasion, radiotherapy resistance, chemotherapy resistance, and recurrence [5,6]. Accumulating evidence has shown that therapeutic resistance to TMZ is primarily driven by GSCs. TMZ treatment consistently increases the GSC population over time in several GBM cell lines and xenografted specimens [7]. Recent clinical observations have revealed that both tumors and recurrent tumors contain a higher proportion of GSCs after chemotherapy and radiotherapy than they contain before therapy [8]. Therefore, the exploration of potential therapeutic strategies for GSC eradication has become an attractive breakthrough in overcoming the therapeutic resistance and recurrence of GBM.

Synthetic lethality, which is the concept that a defect in either one of two genes or proteins has little effect on cells but that simultaneous defects in two genes or proteins with a synthetic lethality interaction lead to cell death, has been studied as a promising therapeutic strategy for targeted cancer therapy [9]. Poly (ADP-ribose) polymerase (PARP) inhibitors, such as talazoparib, olaparib, rucaparib, and niraparib, were the first clinically applied drugs based on synthetic lethality, and they were used in *BRCA1/2*-null ovarian and breast cancers [10]. Synthetic lethality can also be detected using combinations of drugs. Cancer cells are unaffected by the treatment with each drug alone; however, concomitant treatment with two drugs results in synergistic cancer cell death [11]. Chemical synthetic lethality was observed following the combined treatment of ABT-263 and crizotinib targeting BCL-xL/BCL2 and ALK/MET, respectively, in triple-negative breast cancer [12]. Another study revealed that a combination of flutamide and phenprocoumon, which target androgen receptors and vitamin K, respectively, showed synergistic synthetic lethal effects on androgen receptor-positive chronic myeloid leukemia and prostate cancer cells [13]. Accordingly, the identification of synthetic chemical lethality that can strongly suppress the growth of GSCs can lead to the development of promising anticancer therapeutics for GBM.

Calcium signaling is essential for the control of many cellular functions and has been implicated in the onset and maintenance of various diseases, including cancer [14]. Calcium (Ca^2+^)/calmodulin (CaM)-dependent protein kinase II (CaMKII), one of the most important regulators of calcium signaling, is a multifunctional serine/threonine kinase [14]. Accumulating evidence has shown that CaMKII plays a critical role in the survival, proliferation, invasion, and differentiation of various cancer cells by activating multiple signaling pathways, such as the extracellular signal-regulated kinase (ERK), protein kinase B (AKT), the signal transducer and activator of transcription 3 (STAT3), and Wnt/β-catenin signaling pathways [15,16,17]. Furthermore, emerging evidence has revealed a critical role of CaMKII in the survival, proliferation, and maintenance of cancer stem cells [18]. It has been reported that the γ isoform of CaMKII (CaMKIIγ) is required for the maintenance of stem-like and tumorigenic properties in blood, lung, liver, and prostate cancer cells [15,19,20,21]. CaMKIIγ acts as a major molecular switch for regulating the NF-κB, Wnt/β-catenin, Notch, STAT3, and AKT signaling pathways, which are essential for cancer stem-like features in these cancer cells [19,22,23,24]. Moreover, the CaMKIIγ inhibitor berbamine suppresses leukemia stem cells and liver cancer-initiating cells [19,21]. More recently, the synthetic curcumin derivative hydrazinobenzoylcurcumin (HBC), a Ca^2+^/CaM antagonist, inhibited not only the self-renewal capacity but also the metastatic potential of GSCs by blocking the CaM/CaMKII/c-Met signaling pathway [25]. A selective CaMKII inhibitor, KN93, also inhibited the growth of GSCs and the expression of GSC stemness markers [25]. Additionally, CaMKIIγ knockdown decreased the stem-like features of GBM cells [25]. Therefore, CaMKII has attracted attention as an emerging target for eliminating cancer stem cells.

**Figure 1:**
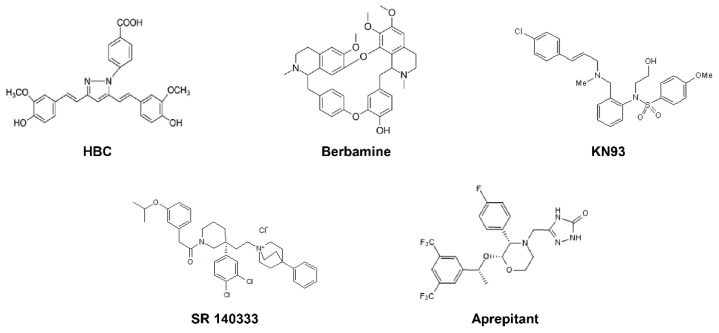
The chemical structures of CaMKII and NK1R inhibitors.

To explore a new CaMKII-targeted synthetic lethal therapy against GSCs, we recently performed high-throughput drug combination screening using CaMKII inhibitors and a bioactive compound library in U87MG- and U373MG-derived GSCs. In the present study, we show that neurokinin 1 receptor (NK1R) inhibitors, such as SR 140333 and aprepitant, have significant chemical synthetic lethal activity against CaMKII inhibitors, including HBC, berbamine, and KN93, in GSCs (Figure 1). The combined treatment of CaMKII and NK1R inhibitors not only markedly decreased GSC viability but also potently suppressed GSC-derived tumor growth in an in vivo model of tumorigenesis. We also identified a synthetic lethal interaction between CaMKII and NK1R using RNA interference. Furthermore, the synergistic anticancer effect of the combination of CaMKII and NK1R inhibitors on GSCs was associated with the downregulation of PI3K/AKT/NF-κB and calcium signaling. These findings demonstrate that NK1R is a potential synthetic lethal partner of CaMKII in eradicating GSCs, and they propose a new CaMKII-targeted combination therapy for GBM treatment.

## 2. Materials and Methods

### 2.1. Materials

Hydrazinobenzoylcurcumin (HBC) was purchased from US Biological (Salem, MA, USA). Berbamine and KN93 were purchased from Sigma-Aldrich (Saint Louis, MO, USA). SR 140333 and aprepitant were obtained from Tocris (Bristol, UK). The compounds were dissolved in dimethyl sulfoxide (DMSO) at a final concentration of 100 mM. The negative control groups were treated with equal volumes of DMSO. DMEM/F12 and Accutase were purchased from HyClone (Marlborough, MA, USA) and EMD Millipore (Temecula, CA, USA), respectively. Epidermal growth factor (EGF) and basic fibroblast growth factor (bFGF) were obtained from Prospecbio (East Brunswick, NJ, USA). B-27 serum-free supplement, L-glutamine, and penicillin/streptomycin were purchased from Gibco (Grand Island, NY, USA). Heparin, intralipid, and 2′,7′-dichlorodihydrofluorescein diacetate (H_2_DCFDA) were purchased from Sigma-Aldrich (Saint Louis, MO, USA). Matrigel was purchased from Corning (Tewksbury, MA, USA). The CellTiter-Glo^®^ 2.0 Cell Viability Assay and Muse^®^ Annexin V & Dead Cell kits were purchased from Promega (Madison, WI, USA) and Luminex (Austin, TX, USA), respectively. Antibodies against CD133 (cat. no. 64326), CD44 (cat. no. 37259), Sox2 (cat. no. 3579), Oct4 (cat. no. 2750), ALDH1A1 (cat. no. 12035), integrin α6 (cat. no. 3750), p53 (cat. no. 9282), cleaved caspase-9 (cat. no. 9501), cleaved caspase-8 (cat. no. 9748), cleaved caspase-3 (cat. no. 9661), cleaved PARP (cat. no. 9542), PI3K (cat. no. 4257), phospho-PI3K (Tyr458, cat. no. 4228), AKT (cat. no. 9272), phospho-AKT (Ser473, cat. no. 4060), NFκB (cat. no. 8242), phospho-NFκB (Ser536, cat. no. 3033), rabbit IgG (cat. no. 7074), and mouse IgG (cat. no. 7076) were purchased from Cell Signaling Technology (Danvers, MA, USA). Anti-CaMKIIγ (cat. no. PA5-29648) and anti-phospho-CaMKIIγ (Thr287, cat. no. PA5-37833) were obtained from Thermo Fisher Scientific (Rockford, IL, USA). Anti-NK1R (cat. no. ab183713) and anti-β-actin (cat. no. ab6276) were obtained from Abcam (Cambridge, UK). Fluo-4 AM ester was purchased from Biotium (Hayward, CA, USA).

### 2.2. GSC Culture

U87MG (KCLB No. 30014) and U373MG (KCLB No. 30017) human GBM cells were purchased from the Korean Cell Line Bank (Seoul, Korea). The identity of the GBM cell lines was confirmed by STR profiling (D3S1358: 16, 17; vWA: 15, 17; FGA: 18, 24; Amelogenin: X, Y; TH01: 9.3; TPOX: 8; CSF1PO: 10, 11; D5S818: 11, 12; D13S317: 8, 11; D7S820: 8, 9 for U87MG; D3S1358: 16, 17; vWA: 16, 18; FGA: 21, 25; Amelogenin: X, Y; TH01: 9.3; TPOX: 8; CSF1PO: 11, 12; D5S818: 11, 12; D13S317: 10, 11; D7S820: 10, 12 for U373MG). To propagate GSCs, the GBM cells were cultured in DMEM/F12 containing 1 × B-27, 5 μg/mL heparin, 2 mM L-glutamine, 20 ng/mL EGF, 20 ng/mL bFGF, and 1% penicillin/streptomycin. Tumorspheres grown in the serum-free media were subcultured every 7 days by dissociating with Accutase. The cells were maintained at 37 °C in a humidified CO_2_ incubator with 5% CO_2_ (Thermo Scientific, Vantaa, Finland).

### 2.3. Cell Viability Assay

Cell viability was quantitatively measured using the CellTiter-Glo^®^ 2.0 Cell Viability Assay kit. Briefly, U87MG- and U373MG-derived GSCs (3 × 10^3^ cells/well) were seeded in 96-white-well culture plates using the serum-free DMEM/F12 media with EGF and bFGF and were treated with the indicated concentrations of each compound. Following incubation for 7 days, each well in the culture plates was treated with 50 μL of substrate solution, shaken for 2 min, and stabilized for 10 min in a dark environment. The luminescence signal was detected using a luminescence reader (BioTek, Inc., Winooski, VT, USA).

### 2.4. Tumorsphere Forming Assay

U87MG- and U373MG-derived GSCs (3 × 10^3^ cells/well) were seeded in 96-well culture plates using the serum-free DMEM/F12 media with EGF and bFGF and were treated with the indicated concentrations of each compound. Following incubation for 7 days, the number of tumorspheres that were >70 µm in diameter was counted under an optical microscope (Olympus, Tokyo, Japan).

### 2.5. Chick Embryo Chorioallantoic Membrane (CAM) Assay

To investigate the effect of compounds on GBM tumorigenesis in vivo, a modified CAM assay was performed. Fertilized chick eggs were incubated in a humidified egg incubator (37 °C, 50% humidity) for 5 days. The eggshell was punched, and the shell membrane was carefully peeled away. U87MG-derived GSCs were harvested and suspended in medium. The cells (1 × 10^6^ cells/egg) were mixed with Matrigel (10 mg/mL, 40 μL/egg) in the absence or presence of compounds (5 μg/egg) and were implanted onto the CAM inside the silicone ring (9 mm inner diameter). After incubation for 7 days, 10% intralipid was injected into the chorioallantois. The tumor formed on the CAM was retrieved, and the tumor weight was measured.

### 2.6. Western Blot Analysis

The cells were lysed using RIPA buffer and were supplemented with protease and phosphatase inhibitors (ATTO, Tokyo, Japan). Equal amounts of protein samples were loaded into 7.5–15% sodium dodecyl sulfate-polyacrylamide gels and were separated by electrophoresis. Then, the proteins separated on the gels were blotted onto polyvinylidene difluoride (PVDF) membranes (EMD Millipore, Hayward, CA, USA). The blots were blocked with 5% skim milk solution at room temperature for 1 h prior to immunolabeling with the primary antibodies against CD133, CD44, Sox2, Oct4, ALDH1A1, integrin α6, p53, cleaved caspase-9, cleaved caspase-8, cleaved caspase-3, cleaved PARP, PI3K, phospho-PI3K, AKT, phospho-AKT, NFκB, phospho-NFκB, CaMKIIγ, phospho-CaMKIIγ, NK1R (dilution 1:2000), and β-actin (1:10,000) overnight at 4 °C. The membranes were washed with Tris-buffered saline containing Tween-20 (TBST) three times and incubated with horseradish peroxidase (HRP)-conjugated secondary antibodies (dilution 1:3000) for 1 h at room temperature. The immunolabeled protein bands were detected using an enhanced chemiluminescence (ECL) reagent (Bio-Rad Laboratories, Hercules, CA, USA) and X-ray film (AGFA, Mortsel, Belgium). The protein bands were quantified using ImageJ software, version 1.5 (NIH). The expression levels were measured as the normalized ratio of each target protein to β-actin.

### 2.7. Analysis of Apoptosis

U87MG- and U373MG-derived GSCs (2 × 10^5^ cells/well) were seeded in 60 mm cell culture dishes using the serum-free DMEM/F12 media with EGF and bFGF and were treated with the indicated concentrations of each compound for 48 h. The cells were collected and washed with phosphate-buffered saline (PBS). After staining with 100 μL of Muse^®^ Annexin V & Dead Cell reagent, the cells were analyzed using a Guava^®^ Muse^®^ Cell Analyzer (MuseSoft_V1.8.0.3; Luminex Corporation, Austin, TX, USA) to detect and quantify cellular apoptosis.

### 2.8. Measurement of Reactive Oxygen Species (ROS)

U87MG- and U373MG-derived GSCs (2 × 10^5^ cells/well) were seeded in 24-well culture plates using the serum-free DMEM/F12 media with EGF and bFGF and were treated with the indicated concentrations of each compound for 12 h. The cells were stained with 10 μM H_2_DCFDA by incubating them for 20 min. Intracellular ROS generation was observed under a fluorescence microscope (Optinity KI-2000F, Korea Lab Tech, Seong Nam, Korea). To quantify the ROS levels, the fluorescence density was measured using ImageJ software, version 1.5 (NIH).

### 2.9. RNA Interference

To knock down the expression of target genes, small interfering RNAs (siRNAs) for CaMKIIγ and NK1R were obtained from Bioneer (Daejeon, Korea) and Santa Cruz Biotechnology (Dallas, TX, USA), respectively. The sequence of CaMKIIγ siRNA was as follows: (sense) 5′-GUAGAGUGCUUACGCAAAU-3′; (antisense) 5′-AUUUGCGUAAGCACUCUAC-3′. NK1R siRNA is a pool of three target-specific 19–25 nt siRNAs (cat. no. sc-36069). Non-targeting scrambled siRNA (cat. no. sc-37007, Santa Cruz Biotechnology) was used as a negative control. Cells were transfected with siRNAs using Lipofectamine^TM^ 2000 Reagent (Invitrogen, NY, USA). The expression of target genes was determined using Western blot analysis.

### 2.10. Measurement of Calcium

U87MG- and U373MG-derived GSCs (2 × 10^5^ cells/well) were seeded in 12-well culture plates using the serum-free DMEM/F12 media with EGF and bFGF and were treated with the indicated concentrations of each compound for 12 h. The cells were stained with 15 μM Fluo-4 AM ester by incubating for 15 min. The fluorescent images were obtained using a fluorescence microscope (Optinity KI-2000F, Korea Lab Tech, Seong Nam, Korea), and the fluorescence intensity was detected at wavelengths of 494/506 nm (excitation/emission) using a multimode microplate reader (BioTek, Winooski, VT, USA).

### 2.11. Statistical Analysis

All experiments were repeated at least three times. The results are expressed as the mean ± standard deviation (SD). Statistical analysis was performed by analysis of variance (ANOVA) with Tukey’s post hoc test using the SPSS 9.0 software (SPSS Inc., Chicago, IL, USA). Statistical significance was considered at *p* < 0.05.

## 3. Results

### 3.1. Combined Treatment of CaMKII and NK1R Inhibitors Increases GSC Lethality

**Figure 2:**
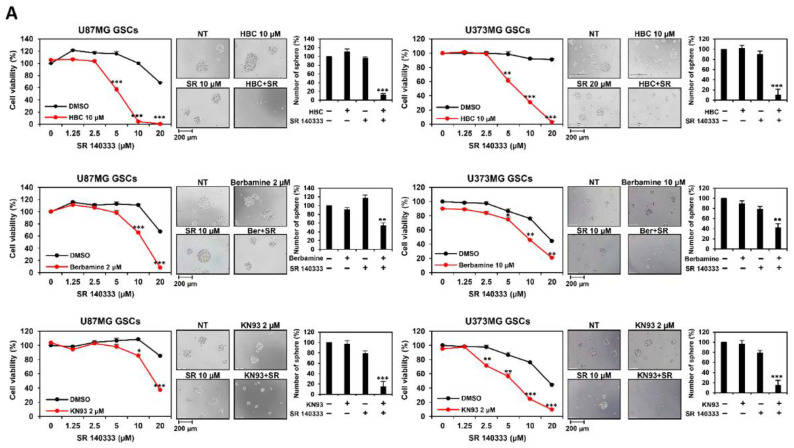

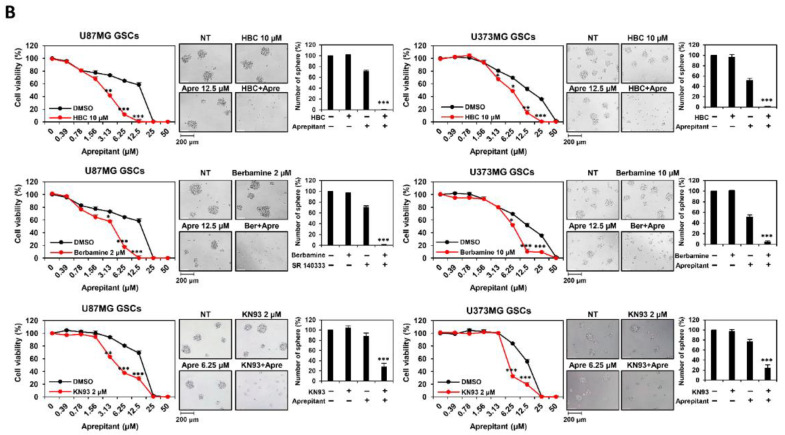
Combined treatment of CaMKII and NK1R inhibitors increases GSC lethality. (**A**,**B**) U87MG- and U373MG-derived GSCs were treated with the indicated concentrations of CaMKII (HBC, Berbamine, KN93) and NKIR inhibitors (SR 140333, Aprepitant) for 7 days. Cell viability was measured using the CellTiter-Glo^®^ luminescent assay system. The number of formed tumorspheres was counted under an optical microscope. SR, SR 140333; Ber, Berbamine; Apre, Aprepitant. * *p* < 0.05, ** *p* < 0.01, *** *p* < 0.001 vs. the compound alone.

To explore a new CaMKII-targeted synthetic lethal therapy against GSCs, high-throughput chemical synthetic lethal screening was performed using 1280 bioactive compounds and CaMKII inhibitors, including HBC, berbamine, and KN93. We found that CaMKII inhibitors with neurokinin 1 receptor (NK1R) inhibitors, such as SR 140333 and aprepitant, significantly increased lethality. As shown in Figure 2A, co-treatment with CaMKII inhibitors and SR 140333 (10 and 20 μM, respectively) resulted in a remarkable reduction in the cell viability and tumorsphere formation of U87MG- and U373MG-derived GSCs compared to single-compound treaments. Furthermore, co-treatment with CaMKII inhibitors and aprepitant (6.25 and 12.5 μM, respectively) markedly suppressed cell viability and tumorsphere formation compared to single treatments (Figure 2B). These results indicate that the combined treatment with CaMKII and NK1R inhibitors exhibits promising anticancer effects against GSCs by causing synthetic lethality.

### 3.2. Combined Treatment of CaMKII and NK1R Inhibitors Potently Suppresses GSC-Derived Tumor Growth In Vivo

**Figure 3:**
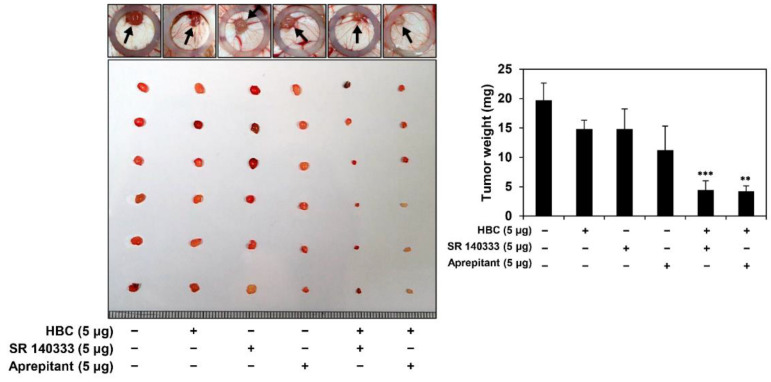
Combined treatment of CaMKII and NK1R inhibitors potently suppressed in vivo tumor growth of GSCs in a CAM model. Fertilized chick eggs were incubated in a humidified incubator at 37 °C. At embryonic day five, U87MG-derived GSCs were mixed with Matrigel in the absence or presence of the indicated compounds (5 μg/egg) and were implanted onto the CAM surface inside the silicone ring. Seven days later, the CAMs were photographed, the formed tumors were retrieved, and the tumor weight was calculated. ** *p* < 0.01, *** *p* < 0.001 vs. the compound alone.

To further assess the effect of the simultaneous treatment with CaMKII and NK1R inhibitors on GBM tumorigenesis by GSCs in vivo, we used a chick chorioallantoic membrane (CAM) tumor model implanted with U87MG GSCs. As shown in Figure 3, the tumor weight in the control group was 19.7 ± 2.9 mg, whereas those in the single-compound treatment were 14.8 ± 1.5, 14.8 ± 3.4, and 11.2 ± 4.1 mg for HBC, SR 140333, and aprepitant, respectively. Notably, the tumor weights by combined administration of HBC with NK1R inhibitors (SR 140333 and aprepitant, respectively) were 4.4 ± 1.6 and 4.2 ± 0.9 mg, respectively, indicating that the combination treatment remarkably suppressed the U87MG GSC-derived tumor growth in comparison with the single-agent treatments. Collectively, these results demonstrate that the combined treatment of CaMKII and NK1R inhibitors can result in chemical synthetic lethality in GSC growth both in vitro and in vivo.

### 3.3. Combined Treatment of CaMKII and NK1R Inhibitors Synergistically Suppresses Expression of GSC Markers

**Figure 4:**
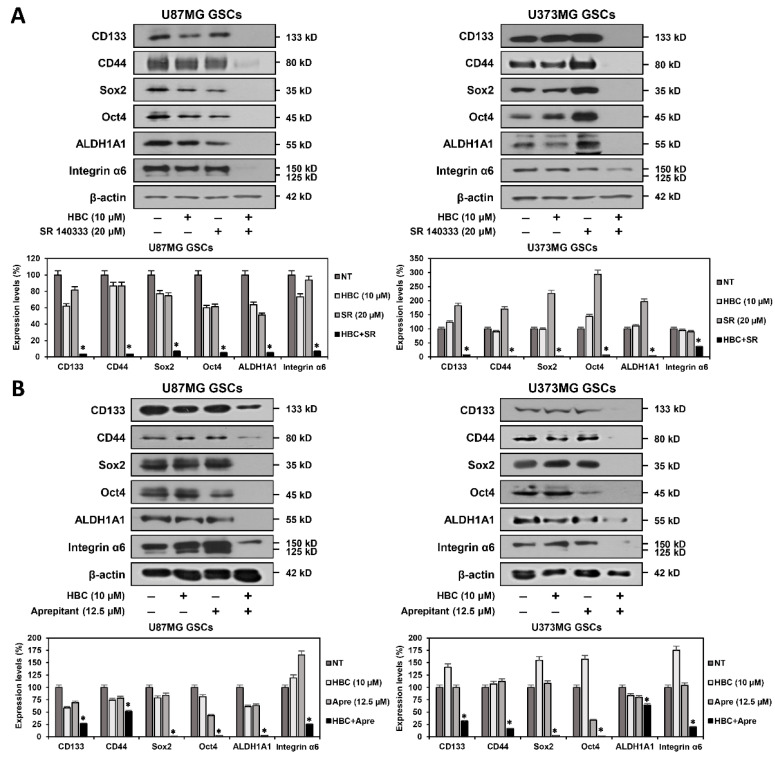
Combined treatment of CaMKII and NK1R inhibitors synergistically suppressed expression of GSC markers. (**A**,**B**) U87MG- and U373MG-derived GSCs were treated with the indicated compounds for 12 h. Protein levels were detected by Western blot analysis using specific antibodies and were further quantified by densitometry. β-actin levels were used as an internal control. Original Western blots are shown in Figure S1. SR, SR 140333; Apre, Aprepitant. * *p* < 0.05 vs. the compound alone.

Several cancer stem cell markers have been implicated in the maintenance of stem-like properties, such as self-renewal and differentiation abilities as well as resistance of GSCs to chemotherapy and radiotherapy [26,27,28]. Therefore, cancer stemness markers represent potentially important therapeutic targets for eliminating GSCs. We investigated whether the synthetic lethal effect of CaMKII and NK1R inhibitors on GSCs was associated with the regulation of the expression of key GSC markers. The combined treatment of HBC and SR 140333 markedly inhibited the expression of the GSC surface glycoproteins CD133 and CD44 compared to single-agent treatments in both U87MG and U373MG GSCs (Figure 4A). In addition, the combination treatment significantly suppressed the expression of the reprogramming transcription factors Oct4 and Sox2 in both GSC lines. Moreover, the simultaneous treatment with HBC and SR 140333 prominently decreased the expression level of aldehyde dehydrogenase 1A1 (ALDH1A1), which is a detoxifying enzyme responsible for the oxidation of intracellular aldehydes and can provide resistance to radiotherapy and chemotherapy of GSCs in both cell types. Furthermore, the combined treatment effectively reduced the expression of the extracellular matrix (ECM) receptor integrin α6, which plays a crucial role in the regulation of stem cell–niche interactions, in comparison with single treatments in both types of GSCs. A synergistic inhibitory effect between CaMKII and NK1R inhibitors on the expression of the major GSC markers was also observed in the combination treatment of HBC and aprepitant (Figure 4B). These results suggest that the lethal effect of CaMKII and NK1R inhibitors on GSCs is related to a potent reduction in the expression of major GSC markers.

### 3.4. Combined Treatment of CaMKII and NK1R Inhibitors Strongly Promotes the Apoptotic Cell Death of GSCs

**Figure 5:**
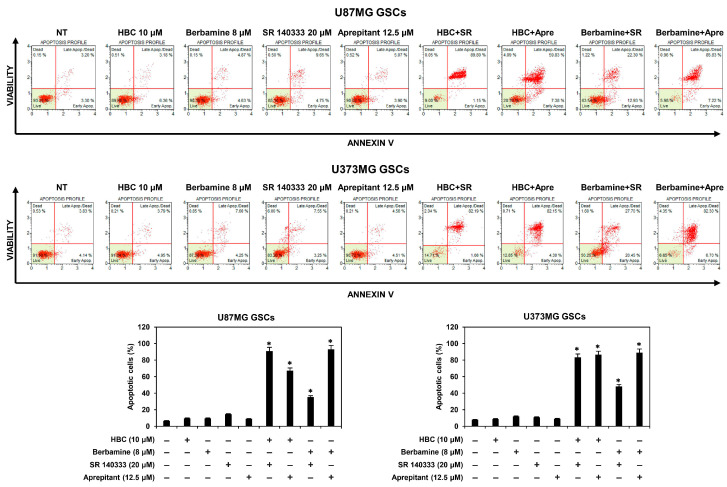
Combined treatment of CaMKII and NK1R inhibitors strongly promoted apoptotic cell death of GSCs. U87MG- and U373MG-derived GSCs were treated with the indicated compounds for 48 h. Apoptotic cells were detected using a Muse Cell Analyzer with Muse^®^ Annexin V & Dead Cell kit. SR, SR 140333; Apre, Aprepitant. * *p* < 0.05 vs. the compound alone.

Cancer stem cells can proliferate and survive abnormally, thereby avoiding apoptosis [29]. Thus, we investigated whether the synthetic lethal effect of CaMKII and NK1R inhibitors on GSCs was associated with apoptotic cell death. Cellular apoptosis was analyzed by flow cytometry using annexin V and propidium iodide (PI) staining. As shown in Figure 5, the combined treatment of CaMKII inhibitors (HBC or berbamine) and NK1R inhibitors (SR 140333 or aprepitant) markedly increased apoptosis in both U87MG and U373MG GSCs, as compared to single-compound treatments. These data demonstrate that the increase in GSC lethality induced by the combination of CaMKII and NK1R inhibitors was caused by the strong promotion of apoptosis in GSCs.

**Figure 6:**
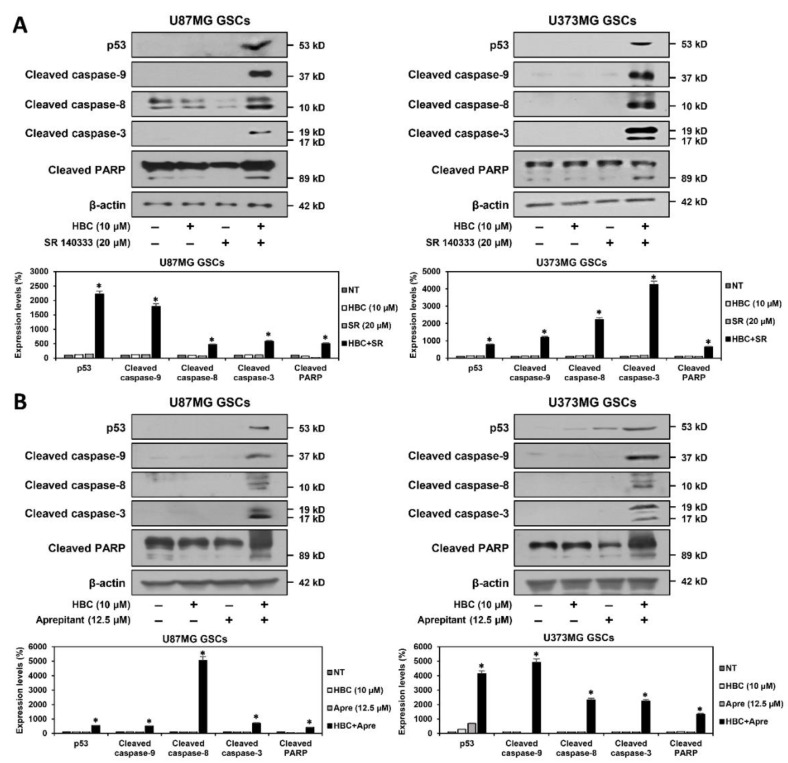
Effect of combined treatment of CaMKII and NK1R inhibitors on the expression of apoptosis regulators in GSCs. (**A**,**B**) U87MG- and U373MG-derived GSCs were treated with the indicated compounds for 48 h. Protein levels were detected by Western blot analysis using specific antibodies and were further quantified by densitometry. β-actin levels were used as an internal control. Original Western blots are shown in Figure S1. SR, SR 140333; Apre, Aprepitant. * *p* < 0.05 vs. the compound alone.

The tumor suppressor gene p53 functions as an upstream regulator of the caspase-mediated apoptotic pathway by inducing the transcriptional activation of proapoptotic genes [30]. Caspases serve as essential effectors of apoptosis and are activated by proteolytic cleavage [31]. Caspase-8 and caspase-9 mediate the extrinsic and intrinsic apoptotic pathways, respectively. These caspases further process caspase-3, a critical executioner of apoptosis. Cleaved caspase-3 is responsible for proteolytic cleavage of downstream substrates involved in apoptotic changes, including poly (ADP-ribose) polymerase (PARP). We further assessed the effect of the combined treatment with CaMKII and NK1R inhibitors on the expression of key mediators of apoptosis in U87MG and U373MG GSCs. The co-treatment with HBC and SR 140333 markedly increased the expression of p53 compared to single-agent treatments in both types of GSCs (Figure 6A). Moreover, the combination of the two compounds significantly upregulated the levels of the cleaved forms of caspase-9, caspase-8, caspase-3, and PARP. The synergistic effect of CaMKII and NK1R inhibitors on these apoptosis markers was also confirmed by the combined treatment with HBC and aprepitant (Figure 6B). These results indicate that the proapoptotic effect of the combined treatment with CaMKII and NK1R inhibitors may result from the increased activation of caspase-mediated apoptotic pathways in GSCs.

### 3.5. Combined Treatment of CaMKII and NK1R Inhibitors Increases ROS Generation in GSCs

**Figure 7:**
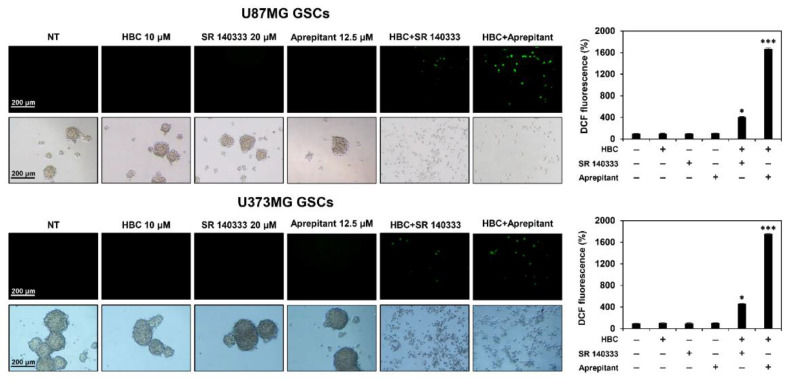
Effect of combined treatment of CaMKII and NK1R inhibitors on the generation of intracellular ROS in GSCs. U87MG and U373MG GSCs were treated with the indicated compounds for 12 h. The levels of ROS were detected with H_2_DCFDA using a fluorescence microscope and were further quantified by densitometry. * *p* < 0.05, *** *p* < 0.001 vs. the compound alone.

Many studies have reported that reactive oxygen species (ROS) play an important role in the induction of apoptosis [31]. To confirm whether simultaneous treatment with CaMKII and NK1R inhibitors affects the generation of ROS in GSCs, the level of intracellular ROS was measured using 2′,7′-dichlorofluorescein diacetate (H_2_DCFDA). The co-treatment with the CaMKII inhibitor HBC and NK1R inhibitor SR 140333 or aprepitant led to a significant increase in the intracellular ROS levels in both U87MG and U373MG GSCs in comparison with the single-compound treatments (Figure 7). Consequently, the accumulation of ROS resulted in GSC death, as observed in the corresponding cell morphology. These findings suggest that the synthetic lethal effect of CaMKII and NK1R inhibitors on GSCs is associated with the synergistic activation of ROS-dependent apoptotic pathways.

### 3.6. Identification of Synthetic Lethal Interaction between CaMKII and NK1R by RNA Interference in GSCs

**Figure 8:**
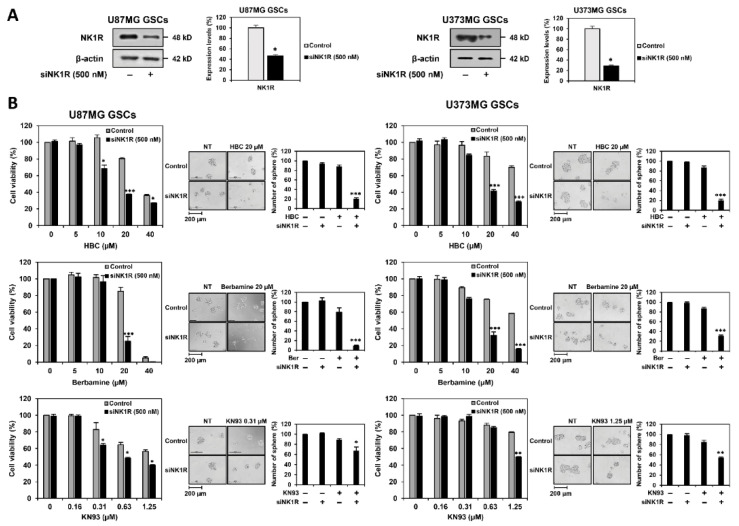

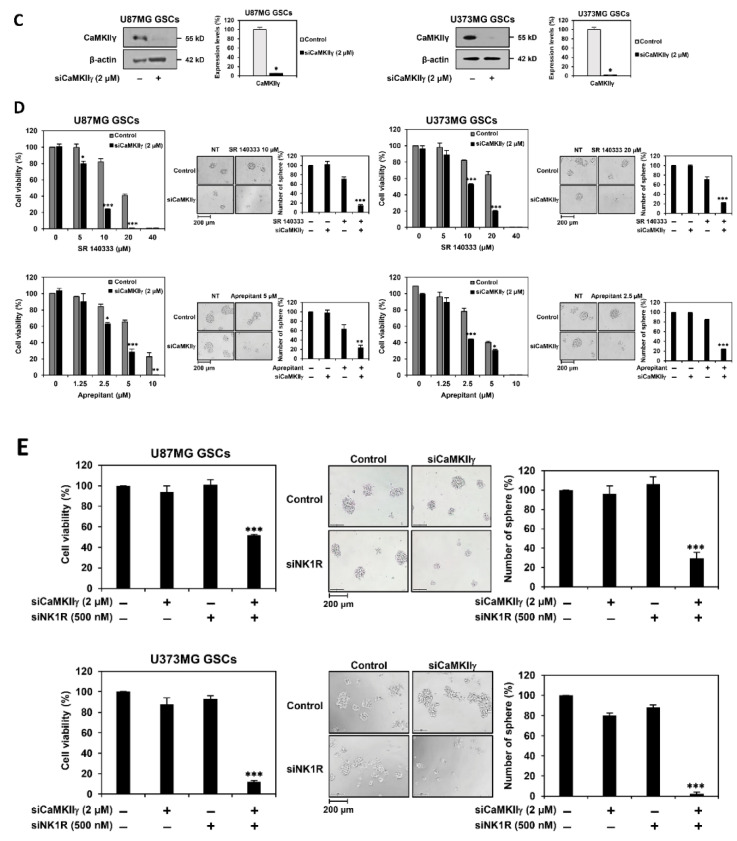
Identification of synthetic lethal interaction between CaMKII and NK1R by RNA interference in GSCs. U87MG and U373MG cells were transfected with either NK1R siRNA or CaMKIIγ siRNA. Knockdown of (**A**) NK1R and (**C**) CaMKIIγ genes was confirmed by Western blot analysis. Original Western blots are shown in Figure S1. * *p* < 0.05 vs. the control siRNA. Following genetic knockdown, U87MG- and U373MG-derived GSCs were treated with the indicated concentrations of (**B**) CaMKII (HBC, Berbamine, KN93) inhibitors and (**D**) NK1R inhibitors (SR 140333, Aprepitant) for 7 days. Cell viability was measured using the CellTiter-Glo^®^ luminescent assay system. The number of formed tumorspheres was counted under an optical microscope. (**E**) The effect of simultaneous knockdown of CaMKIIγ and NK1R genes on the cell viability and tumorsphere formation of both GSCs was also determined after incubation for 7 days. * *p* < 0.05, ** *p* < 0.01, *** *p* < 0.001 vs. the compound alone or the single gene knockdown.

To clarify whether the synergistic effect of CaMKII and NK1R inhibitors in eradicating GSCs resulted from the synthetic lethal interaction between CaMKII and NK1R genes, we performed genetic knockdown experiments using small interfering RNAs (siRNAs) targeting either CaMKIIγ or NK1R. First, U87MG and U373MG cells were transfected with either human NK1R-specific siRNA (siNK1R) or control siRNA, and NK1R silencing was confirmed by Western blotting (Figure 8A). Following NK1R knockdown, U87MG- and U373MG-derived GSCs were treated with CaMKII inhibitors, including HBC, berbamine, and KN93. The cell viability and tumorsphere formation of both GSCs were more effectively suppressed by the concomitant silencing of the NK1R gene compared to the single treatment with CaMKII inhibitors (Figure 8B). These data imply that the loss of NK1R function may increase the chemosensitivity of GSCs to CaMKII inhibitors.

Next, U87MG and U373MG cells were transfected with either human CaMKIIγ-specific siRNA (siCaMKIIγ) or control siRNA, and the reduced expression of CaMKIIγ was confirmed by Western blotting (Figure 8C). NK1R inhibitors, including SR 140333 and aprepitant, more potently inhibited the cell viability and tumorsphere formation of CaMKIIγ-silenced U87MG and U373MG GSCs compared to non-silenced GSCs (Figure 8D). These data indicate that the downregulation of CaMKIIγ can improve the anticancer effect of NK1R inhibitors against GSCs.

We also confirmed the effect of the simultaneous knockdown of CaMKIIγ and NK1R genes on GSCs. The concurrent silencing of both genes resulted in a remarkable reduction in cell viability and tumorsphere formation of both U87MG- and U373MG-derived GSCs in comparison with the silencing of each gene alone (Figure 8E). These results suggest that NK1R is a synthetic lethal partner of CaMKIIγ, thereby indicating that the combined treatment with CaMKII and NK1R inhibitors exhibits an excellent synergistic effect in eliminating GSCs.

### 3.7. Inhibition of PI3K/AKT/NF-κB and Calcium Signaling Contributes to Synthetic Lethality between CaMKII and NK1R Inhibitors in GSCs

**Figure 9:**
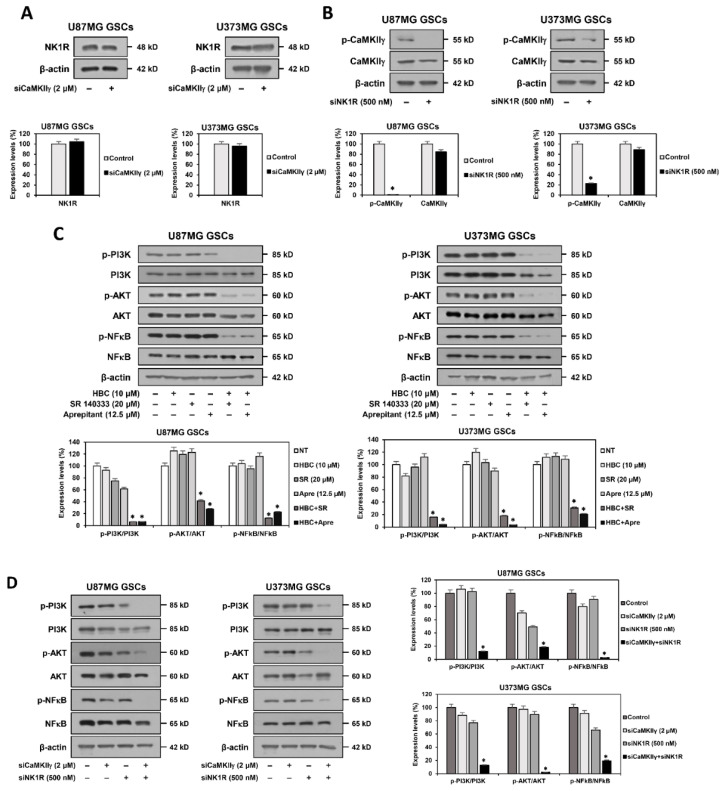

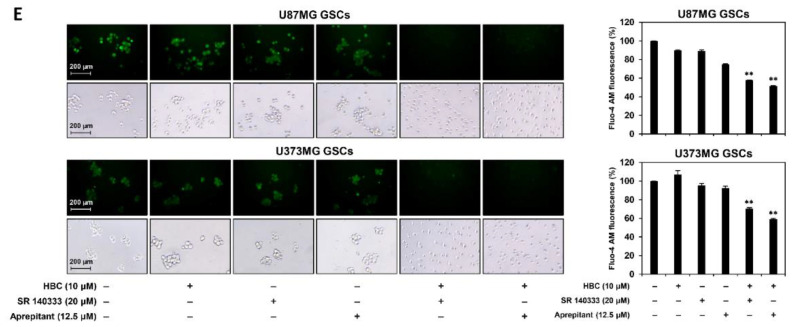
Synthetic lethal effect between CaMKII and NK1R inhibitors on GSCs is associated with the inhibition of PI3K/AKT/NF-κB and calcium signaling. (**A**) Effect of CaMKIIγ knockdown on NK1R expression in U87MG- and U373MG-derived GSCs. (**B**) Effect of NK1R knockdown on CaMKIIγ expression in both GSCs. *p* < 0.05 vs. the control siRNA. (**C**) Effect of combined treatment (12 h) of CaMKII and NK1R inhibitors on PI3K/AKT/NF-κB pathway in both GSCs. (**D**) Effect of concurrent knockdown of CaMKIIγ and NK1R on PI3K/AKT/NF-κB pathway in both GSCs. (**A**–**D**) Protein levels were detected by Western blot analysis using specific antibodies and were further quantified by densitometry. β-actin levels were used as an internal control. Original Western blots are shown in Figure S1. (**E**) Effect of combined treatment (12 h) of CaMKII and NK1R inhibitors on intracellular calcium level in both GSCs. The levels of calcium were detected with Fluo-4 AM using a fluorescence microscope and were further quantified using a multimode microplate reader. * *p* < 0.05, ** *p* < 0.01 vs. the compound alone or the single gene knockdown.

To further elucidate the synthetic lethal mechanism between CaMKII and NK1R, we evaluated whether CaMKIIγ silencing affects NK1R expression in GSCs. As shown in Figure 9A, CaMKIIγ knockdown did not cause a significant change in the expression level of NK1R in either U87MG or U373MG GSCs. However, NK1R silencing did not affect the total level of CaMKIIγ, but rather it reduced its phosphorylation (Figure 9B). These results suggest that NK1R functions as an upstream regulator of CaMKIIγ activity.

Recent studies have revealed that the inhibition of NK1R suppresses cell proliferation and induces apoptosis in the stem-like cells of several cancers, such as esophageal squamous cell carcinoma (ESCC), by blocking the phosphoinositide 3-kinase (PI3K)/protein kinase B (AKT)/nuclear factor-kappa B (NF-κB) pathway [32]. Furthermore, it has been reported that the inhibition of CaMKIIγ reduces the self-renewal ability of GSCs by downregulating the AKT-mediated signaling pathway [25]. Therefore, we assessed whether the combined treatment with CaMKII and NK1R inhibitors affects the PI3K/AKT/NF-κB pathway. As shown in Figure 9C, co-treatment with the CaMKII inhibitor HBC and NK1R inhibitor SR 140333 or aprepitant effectively suppressed the phosphorylation of PI3K, AKT, and NF-κB compared to single-compound treatments in both U87MG and U373MG GSCs. Moreover, the synergistic effect of the compounds was further verified by concurrent silencing of the corresponding target genes. Our results show that the knockdown of both CaMKIIγ and NK1R genes markedly inhibited the phosphorylation of PI3K, AKT, and NF-κB in both GSCs in comparison with gene silencing alone (Figure 9D). These results imply that the synthetic lethal effect of CaMKII and NK1R inhibitors on GSCs may be involved in the blockade of the PI3K/AKT/NF-κB signaling cascade.

Intracellular calcium concentrations are important for the regulation of CaMKII activity [33]. We further investigated whether the combined treatment with CaMKII and NK1R inhibitors affects the intracellular calcium levels of GSCs using the fluorescent calcium indicator Fluo-4 AM. As shown in Figure 9E, the intracellular calcium concentration was significantly decreased by the simultaneous treatment with the CaMKII inhibitor HBC and the NK1R inhibitor SR 140333 or aprepitant, compared to single-compound treatments in both the U87MG and U373MG GSCs. Collectively, these results suggest that the considerable inhibition of PI3K/AKT/NF-κB and calcium signaling may play a critical role in inducing the synthetic lethality between CaMKII and NK1R inhibitors on GSCs.

## 4. Discussion

CaMKII is a multifunctional serine/threonine-protein kinase consisting of four homologous isoforms (CaMKIIα/β/γ/δ) and is involved in diverse cellular processes [34]. Accumulating evidence highlights the involvement of CaMKII in cancer development [15]. The pharmacological inhibition of CaMKII by several specific inhibitors, such as KN62 and KN93, suppresses cancer cell malignancies, including cell survival, proliferation, migration, and invasion, by promoting cell cycle arrest and cell apoptosis [35]. In addition, CaMKII has been implicated in the activation of multiple oncogenic signaling pathways, such as the MAPK, AKT/mTOR, JAK/STAT, GSK3β/β-catenin, Notch, and NF-κB pathways, which play a critical role in cancer progression [15,22,23,24]. Furthermore, several recent studies have revealed the emerging role of CaMKIIγ in the maintenance of the stem-like traits of cancer cells, which drive tumor initiation, metastasis, drug resistance, and recurrence [19,20,22]. CaMKIIγ enhanced the stem-like features and tumorigenicity of lung cancer cells, including Oct4 expression and tumorsphere formation, in an AKT-dependent and β-catenin-dependent manner [20]. CaMKIIγ is also highly activated in leukemia stem/progenitor cells and promotes cell survival and self-renewal by activating β-catenin, NF-κB, and STAT3 signaling [19]. Notably, berbamine, which specifically binds to the ATP-binding pocket of CaMKIIγ and inhibits its kinase activity, suppresses the growth of leukemia stem cells via the downregulation of these signaling pathways [19]. Berbamine also inhibits the self-renewal ability of liver cancer stem cells by targeting CaMKIIγ [21]. More recently, our study demonstrated that the synthetic curcumin derivative HBC, a Ca^2+^/CaM antagonist, inhibits the stem-like features of GBM cells by downregulating the CaM/CaMKIIγ/c-Met pathway [25]. Moreover, genetic knockdown of CaMKIIγ decreased the cancer stem-like traits of GBM cells, such as neurosphere formation and the expression of stemness markers [25]. Therefore, these findings indicate that CaMKIIγ is a promising therapeutic target for eradicating cancer stem cells, including GSCs.

In the current study, we aimed to explore a new CaMKII-targeted combination therapy for GSCs using a chemical synthetic lethal approach. Through high-throughput drug combination screening using CaMKII inhibitors and a bioactive compound library in GSCs, neurokinin 1 receptor (NK1R) inhibitors such as SR 140333 and aprepitant were found to be potential anticancer agents that significantly increased GSC lethality by co-treatment with CaMKII inhibitors, including HBC, berbamine, and KN93. Combined treatment with CaMKII and NK1R inhibitors not only markedly suppressed GSC viability and tumorsphere formation but also effectively inhibited GSC-derived tumor growth in comparison with a single-compound treatment in an in vivo model of tumorigenesis. In addition, the lethal effect of CaMKII and NK1R inhibitors on GSCs was associated with a remarkable reduction in the expression of key GSC markers, including CD133, CD44, Sox2, Oct4, ALDH1A1, and integrin α6. Notably, the synergistic anticancer effect of CaMKII and NK1R inhibitors against GSCs was caused by the promotion of ROS-dependent apoptosis through strong activation of the caspase cascade mediated by p53. Furthermore, we verified the synthetic lethal interaction between CaMKII and NK1R by using siRNAs targeting either CaMKIIγ or NK1R. Taken together, our findings demonstrate for the first time that NK1R is a potential synthetic lethal partner of CaMKII to eradicate GSCs, and therefore, they suggest a new combination therapy targeting CaMKIIγ and NK1R for GBM treatment.

NK1R, also known as the substance P (SP) receptor, is a G protein-coupled receptor that regulates a wide range of biological functions, mainly those in the central and peripheral nervous systems [36,37]. The activation of NK1R induces neurogenic inflammation, chemotherapy-induced nausea and vomiting (CINV), and multiple oncogenic signaling cascades [38]. NK1R plays a crucial role in cancer development by regulating cell survival, proliferation, migration, invasion, and angiogenesis [39]. NK1R is overexpressed in many human cancers, including GBM, and its increased expression is strongly associated with higher tumor malignancy and a poor prognosis [40]. NK1R stimulation induces GBM cell proliferation through activation of the ERK and AKT pathways [38]. NK1R also mediates GBM cell migration by upregulating matrix metalloproteinase-2 (MMP-2) and membrane type 1-matrix metalloproteinase (MT1-MMP) [41]. In addition, NK1R agonists, including SP and hemokinin-1 (HK-1), promote GBM cell proliferation and migration, whereas NK1R antagonists, such as aprepitant, effectively inhibit GBM cell growth in vitro and in vivo [42,43]. Aprepitant is a specific NK1R antagonist approved by the US FDA for the prevention of CINV [44]. The combined treatment with aprepitant and maraviroc, an FDA-approved anti-HIV drug that antagonizes CCR5, exhibits a synergistic inhibitory effect on GBM growth [44]. It is worth noting that aprepitant is a drug included in the coordinated undermining of survival path 9 (CUSP9) treatment protocol, a new clinical combination concept to treat patients with recurrent GBM [45]. Notably, the nine repurposed drugs combined with TMZ more efficiently suppress the viability of patient-derived GSCs than the drugs do individually [46]. Recently, the anticancer activity of aprepitant in cancer stem cells has been observed in different cancers. Aprepitant promoted caspase-dependent apoptosis and G2/M cell cycle arrest in ESCC stem-like cells by downregulating the PI3K/AKT/NF-κB pathway [47]. Therefore, these previous reports indicate that NK1R plays a critical role in GBM tumor growth and development. In addition, NK1R positively regulates the growth of cancer stem cells, including GSCs. Accordingly, targeting NK1R can provide an effective therapeutic approach for GBM treatment.

**Figure 10:**
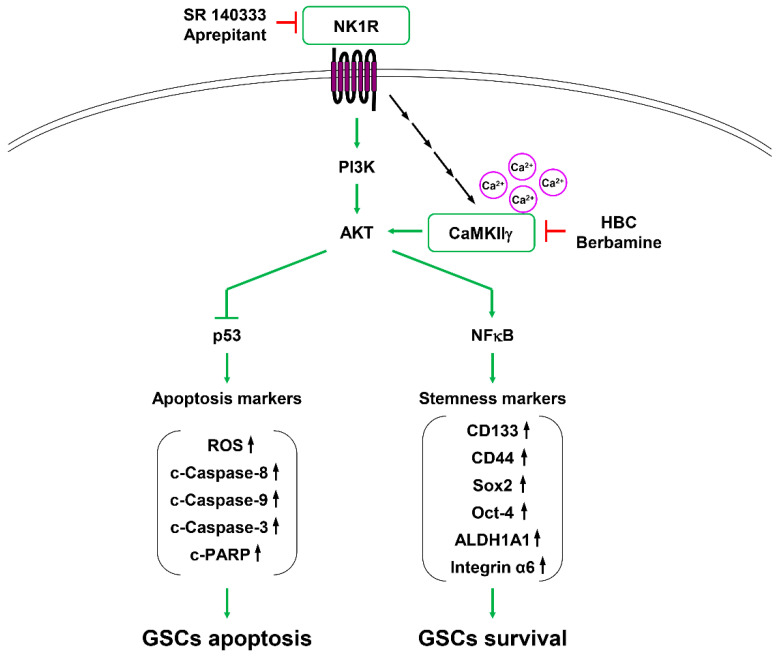
Proposed molecular mechanism of the synthetic lethal effect of CaMKII and NK1R inhibitors on GSCs.

Although aprepitant exerts a combination effect with several targeted anticancer drugs, further exploration of a novel combination partner that shows potent synthetic lethal interaction with NK1R inhibitors can contribute to overcoming chemotherapy resistance and GBM recurrence. Our present study found that the combined treatment with CaMKII and NK1R inhibitors significantly increased GSC lethality, thereby suggesting a new combination therapy targeting CaMKII and NK1R to eliminate GSCs. Furthermore, we elucidated the underlying synthetic lethal mechanism between CaMKII and NK1R (Figure 10). The activation of PI3K/AKT signaling downregulates the function of p53, whereas it upregulates the activity of NF-κB, resulting in an increase in the self-renewal ability of GSCs [48]. Combined inhibition of CaMKIIγ and NK1R strongly blocked the activation of PI3K/AKT signaling. Consequently, the synergistic deactivation of the PI3K/AKT pathway significantly induced the upregulation of p53 and the downregulation of NF-κB and subsequently led to GSC apoptosis by the activation of the caspase cascade, as well as to the reduction in GSC survival by inhibiting the expression of stemness markers. In addition, our results show that NK1R may act as an upstream regulator of CaMKIIγ activity. It has been reported that NK1R activation stimulates phospholipase C and thus induces intracellular calcium release [49]. In the present study, NK1R silencing reduced the phosphorylation of CaMKIIγ, and single treatment with NK1R inhibitors or in combination with the CaMKII inhibitor HBC caused a decrease in the intracellular calcium level. These results imply that NK1R may regulate CaMKIIγ activity by mediating intracellular calcium release and that the inhibition of calcium signaling may partly affect the synthetic lethal effect between CaMKII and NK1R inhibitors in GSCs. These findings demonstrate that NK1R is a potential synthetic lethal partner of CaMKII to eradicate GSCs, and they suggest that the combination treatment of CaMKII and NK1R inhibitors can be utilized as a powerful therapeutic strategy to overcome resistance to chemotherapy and GBM recurrence. Further in vivo studies using animal models are needed to support the chemical synthetic lethal effect.

## 5. Conclusions

In this study, we explored a novel CaMKII-targeted combination therapy to effectively eradicate GSCs using high-throughput chemical synthetic lethal screening. NK1R inhibitors, including SR 140333 and aprepitant, were found to be potential anticancer agents that exhibit a synergistic effect impeding the growth of GSCs in combination with CaMKII inhibitors, such as HBC, berbamine, and KN93. The synthetic lethal effect between CaMKII and NK1R inhibitors on GSCs resulted from the activation of caspase-mediated apoptosis by inducing p53 expression and ROS generation as well as by suppressing the expression of stemness markers through deactivation of NF-κB following the downregulation of the PI3K/AKT pathway and calcium signaling. In addition, the synthetic lethal interaction between CaMKIIγ and NK1R in GSCs was demonstrated by gene silencing using siRNAs. These findings suggest a new combination therapy targeting CaMKIIγ and NK1R for GBM treatment.

## Data Availability

The data that support the findings of this study are available from the corresponding author upon reasonable request.

## Supplementary Materials

The following supporting information can be downloaded at: https://www.mdpi.com/article/10.3390/cancers14051315/s1, Figure S1: original Western blots.
