# Supplementary material for: Discovery of a New CaMKII-Targeted Synthetic Lethal Therapy against Glioblastoma Stem-like Cells
Source: Cancers (Basel). 2022 Mar 4;14(5):1315. doi: 10.3390/cancers14051315 (PMC8909660; doi:10.3390/cancers14051315)
Supplement: Supplementary file 1 [file cancers-14-01315-s001.zip › cancers-1606100-supplementary.pdf]

cancers

MDPI

Supplementary Materials: Discovery of a New CaMKII-Targeted Synthetic Lethal Therapy against Glioblastoma Stem-Like Cells

Jang Mi Han, Yu Jin Kim and Hye Jin Jung

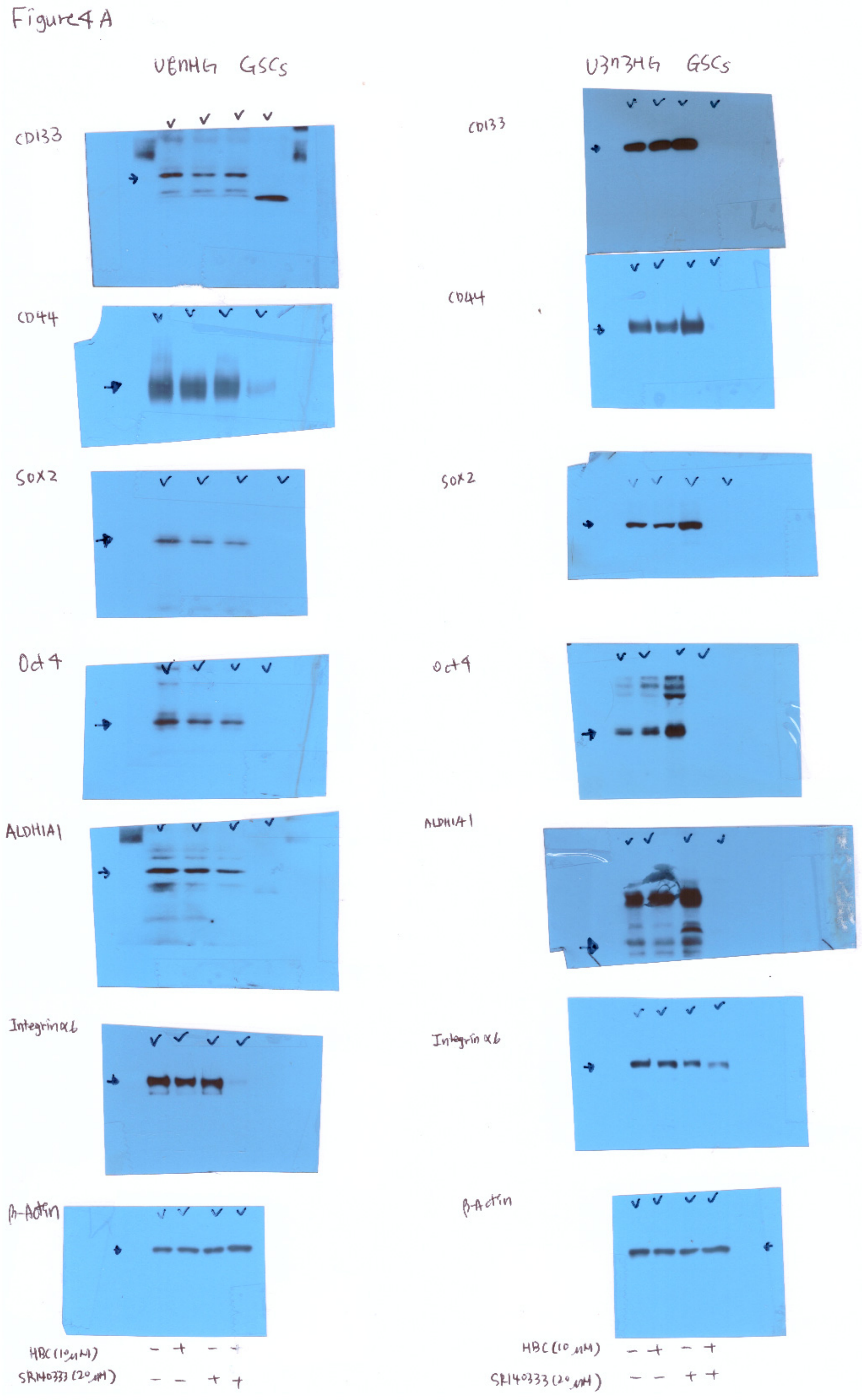
Figure4 A
U87MG GSCs
U373MG GSCs
CD133
CD44
SOX2
Oct4
ALDH1A1
Integrinα6
β-Actin
HBC (10 μM) − + − +
SR140333 (20 μM) − − + +

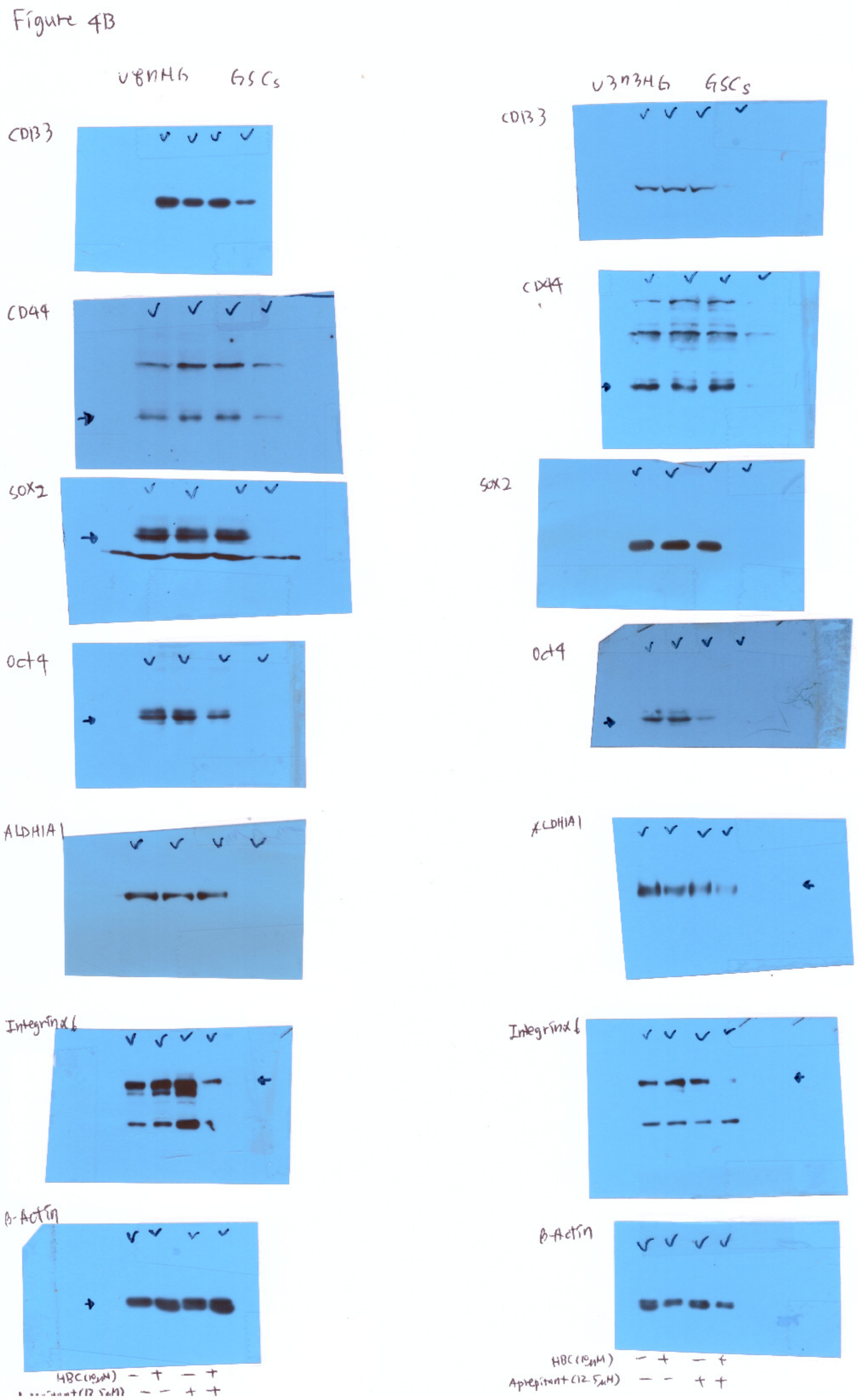
Figure 4B
U87MG GSCs
U373MG GSCs
CD133
CD44
SOX2
Oct4
ALDH1A1
Integrin α6
β-Actin
HBC (10 μM) − + − +
Aprepitant (12.5 μM) − − + +

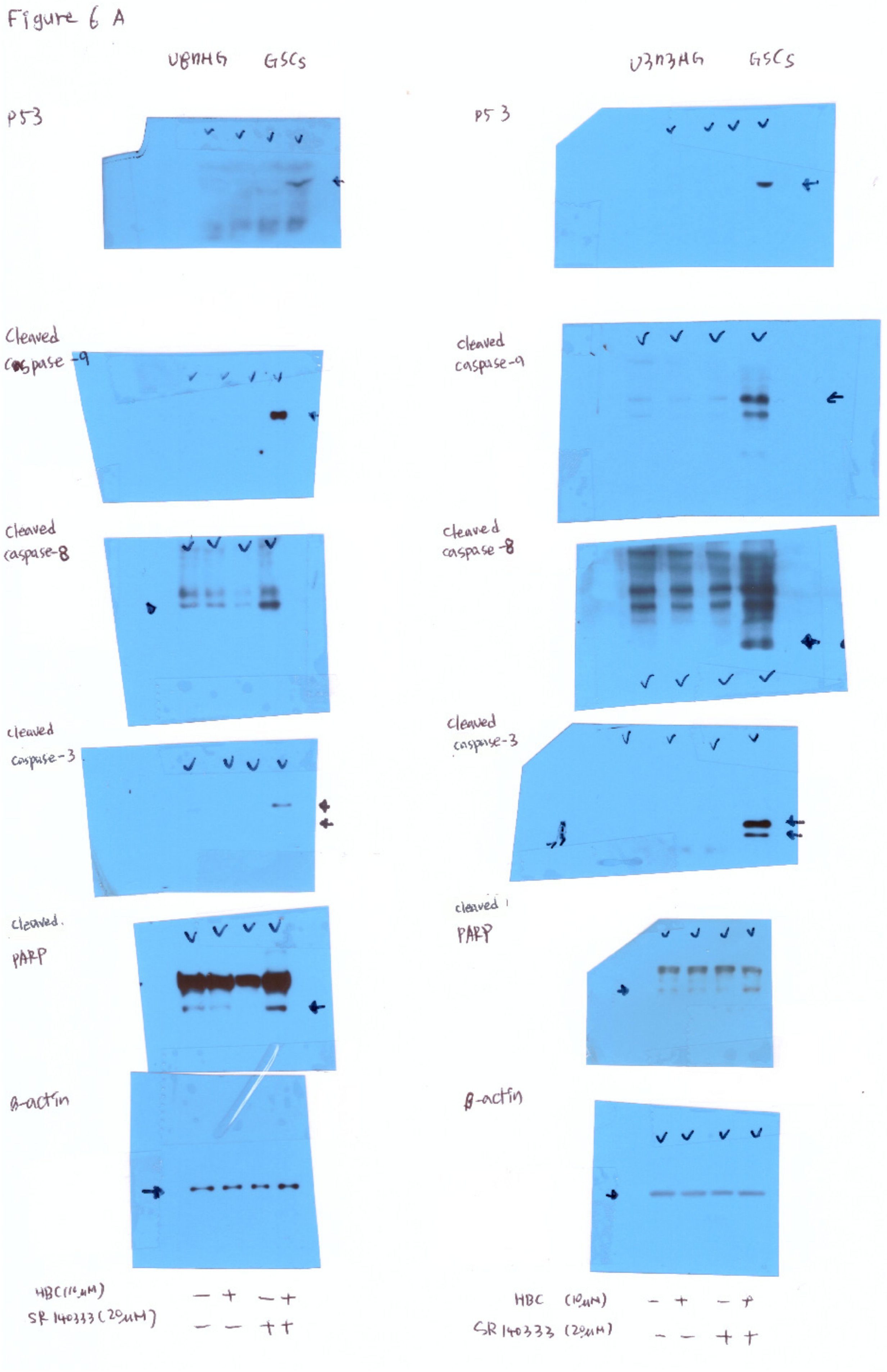
Figure 6 A
U87MG
GSCs
U373MG
GSCs
p53
p53
Cleaved caspase-9
cleaved caspase-9
Cleaved caspase-8
cleaved caspase-8
cleaved caspase-3
cleaved caspase-3
cleaved PARP
cleaved PARP
β-actin
β-actin
HBC (10 μM)
SR 140333 (20 μM)
HBC (10 μM)
SR 140333 (20 μM)

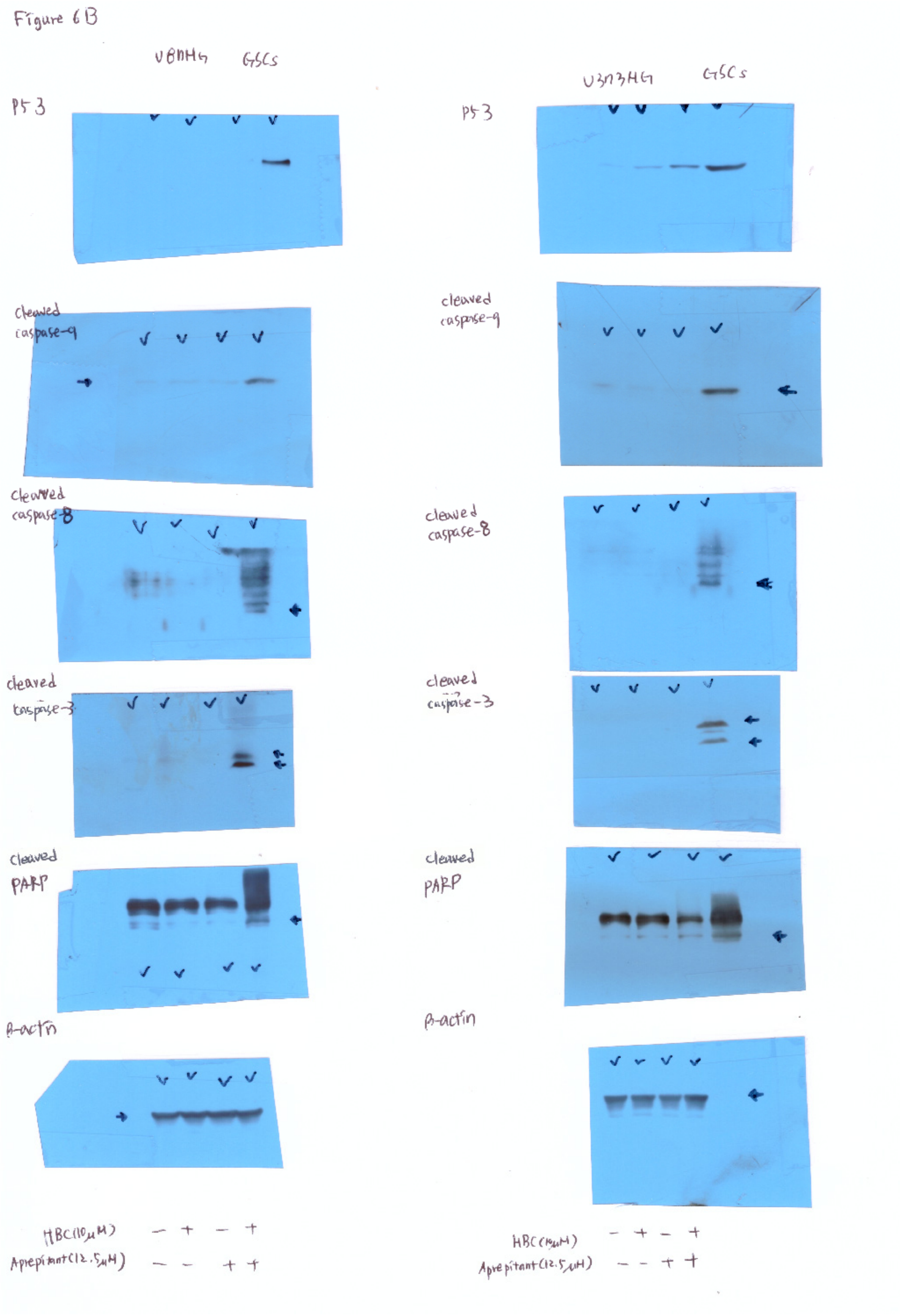
Figure 6B
GSCs
U373MG
GSCs
P53
P53
cleaved caspase-9
cleaved caspase-9
cleaved caspase-8
cleaved caspase-8
cleaved caspase-3
cleaved caspase-3
cleaved PARP
cleaved PARP
β-actin
β-actin
HBC (10 μM)
Aprepitant (12.5 μM)
HBC (10 μM)
Aprepitant (12.5 μM)

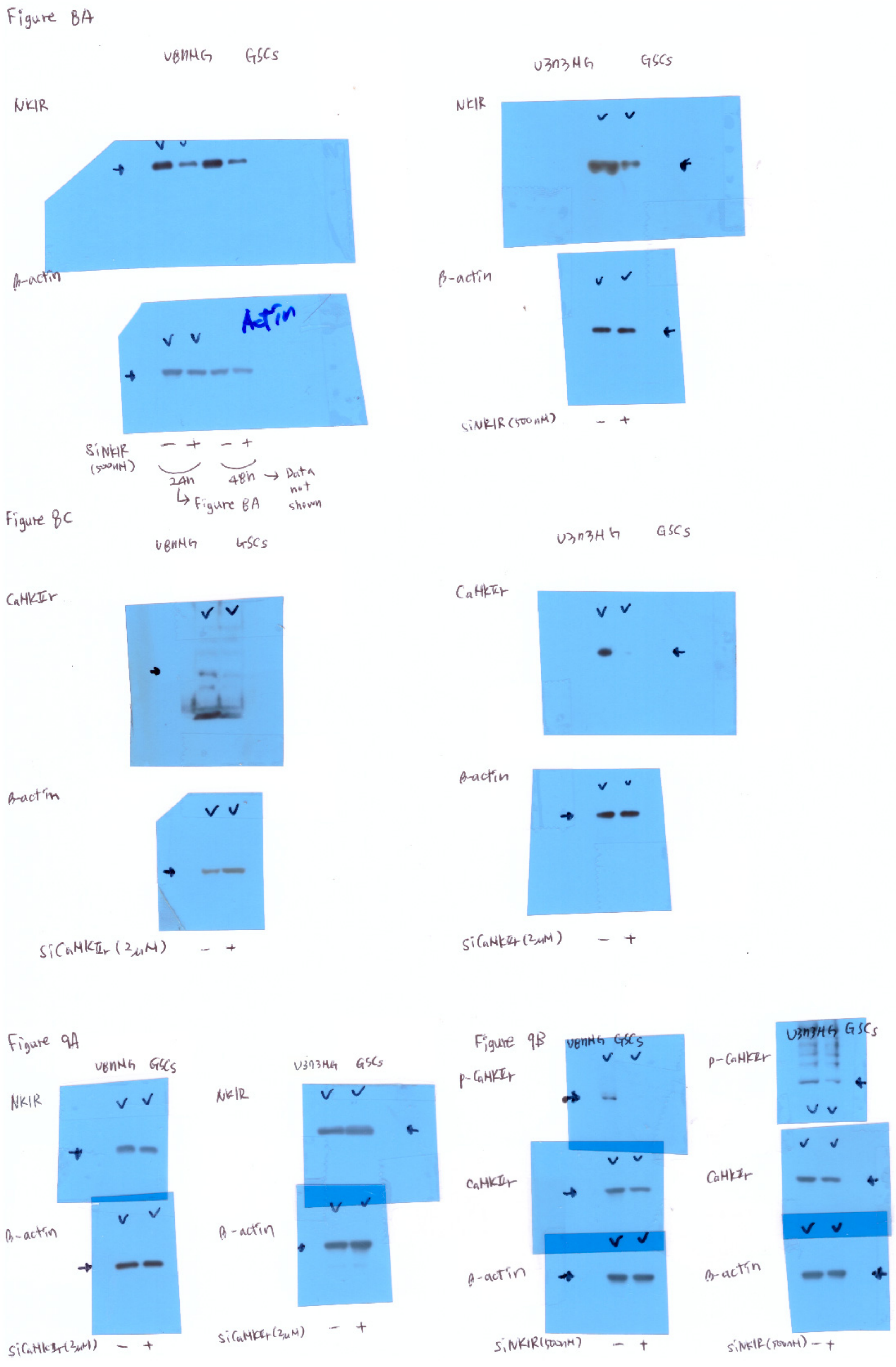
Figure 8A
U87MG
GSCs
NKIR
β-actin
Actin
SiNKIR (500nM)
24h
48h
Data not shown
Figure 8A
U373MG
GSCs
siNKIR (500nM)
Figure 8C
CaMKIIr
SiCaMKIIr (2uM)
Figure 9A
Figure 9B
p-CaMKIIr
siNKIR (500nM)

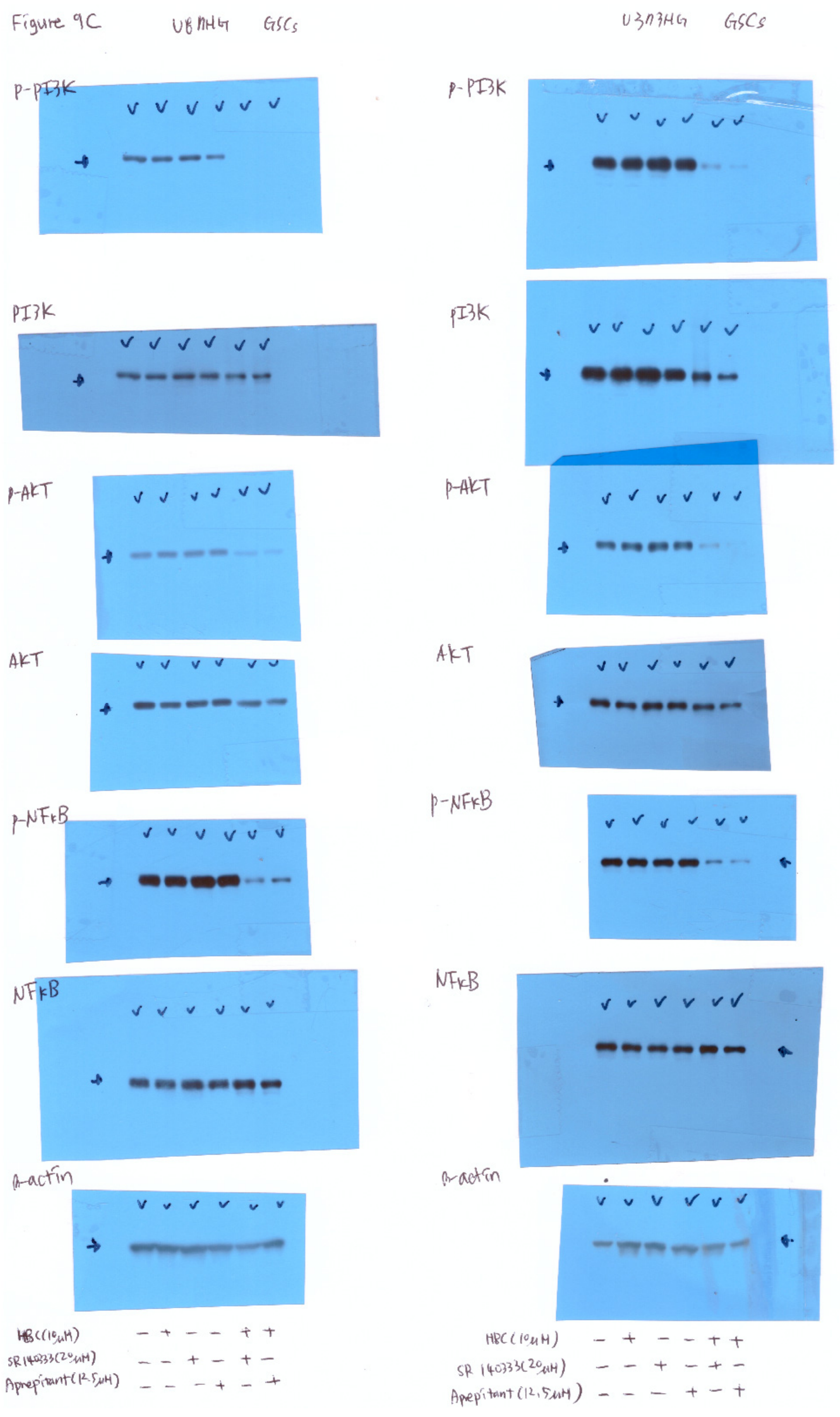
Figure 9C
U87MG GSCs
U373MG GSCs
p-PI3K
PI3K
p-AKT
AKT
p-NFκB
NFκB
β-actin
HBC (10 μM)
SR 140333 (20 μM)
Aprepitant (12.5 μM)

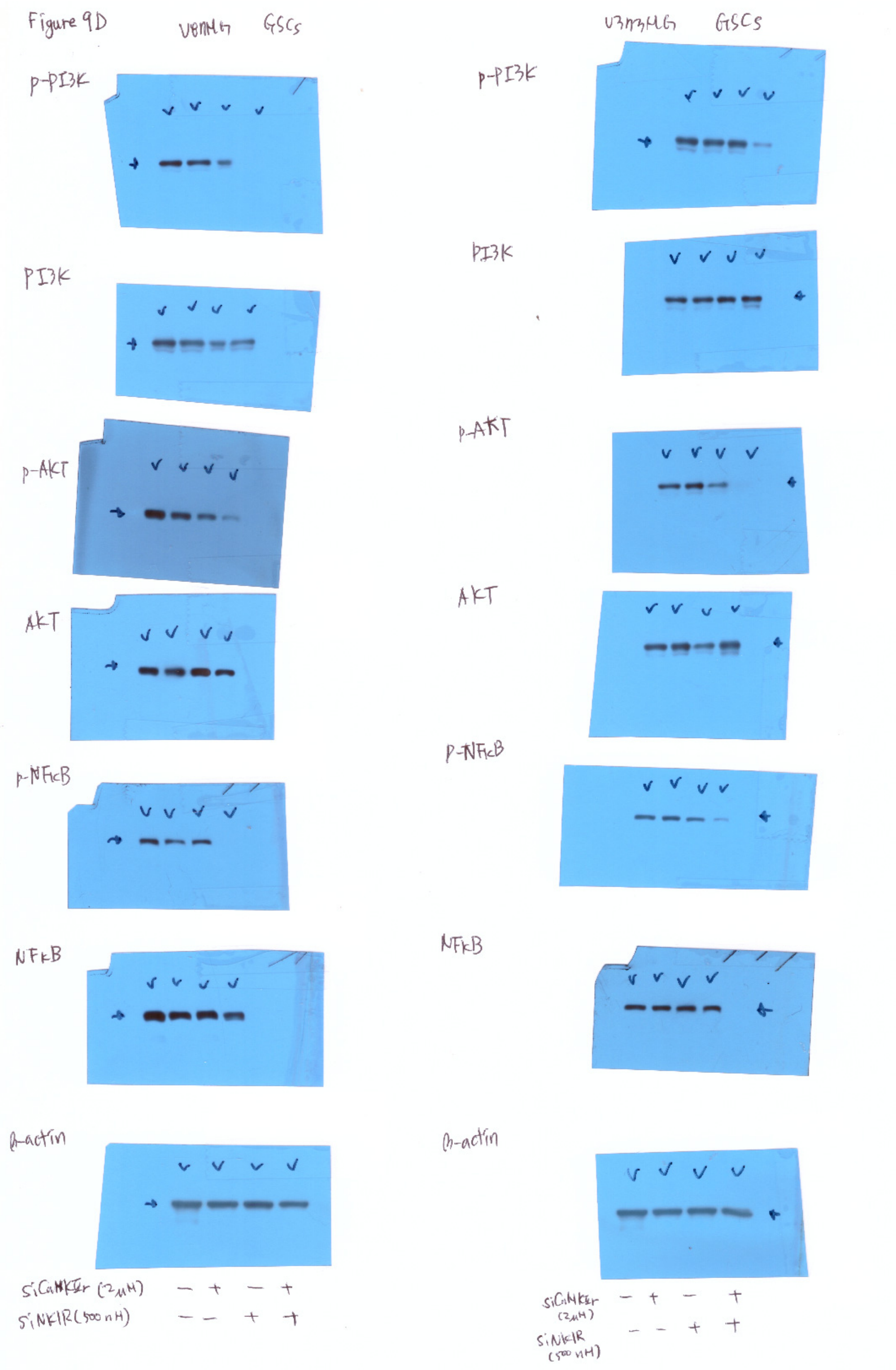

Figure S1. Original western blots.
